# Interference recommendation for the pump sizing process in progressive cavity pumps using graph neural networks

**DOI:** 10.1038/s41598-023-43972-4

**Published:** 2023-10-06

**Authors:** Leandro Starke, Aurélio Faustino Hoppe, Andreza Sartori, Stefano Frizzo Stefenon, Juan Francisco De Paz Santana, Valderi Reis Quietinho Leithardt

**Affiliations:** 1 Department of Information Systems and Computing, Regional University of Blumenau, Rua Antônio da Veiga 140, 89030-903 Blumenau, SC Brazil; 2 Electrical Engineering Graduate Program, Regional University of Blumenau, Rua São Paulo 3250, 89030-000 Blumenau, SC Brazil; 3 Fondazione Bruno Kessler, Via Sommarive 18, 38123 Trento, TN Italy; 4 University of Udine, Via delle Scienze 206, 33100 Udine, UD Italy; 5 Expert Systems and Applications Lab, Faculty of Science, University of Salamanca, 37008 Salamanca, Spain; 6 Instituto Superior de Engenharia de Lisboa (ISEL), Instituto Politécnico de Lisboa, Rua Conselheiro Emídio Navarro, 1, 1959-007 Lisbon, Portugal

**Keywords:** Electrical and electronic engineering, Energy infrastructure, Mechanical engineering

## Abstract

Pump sizing is the process of dimensional matching of an impeller and stator to provide a satisfactory performance test result and good service life during the operation of progressive cavity pumps. In this process, historical data analysis and dimensional monitoring are done manually, consuming a large number of man-hours and requiring a deep knowledge of progressive cavity pump behavior. This paper proposes the use of graph neural networks in the construction of a prototype to recommend interference during the pump sizing process in a progressive cavity pump. For this, data from different applications is used in addition to individual control spreadsheets to build the database used in the prototype. From the pre-processed data, complex network techniques and the betweenness centrality metric are used to calculate the degree of importance of each order confirmation, as well as to calculate the dimensionality of the rotors. Using the proposed method a mean squared error of 0.28 is obtained for the cases where there are recommendations for order confirmations. Based on the results achieved, it is noticeable that there is a similarity of the dimensions defined by the project engineers during the pump sizing process, and this outcome can be used to validate the new design definitions.

## Introduction

In 1933, the French mathematician and researcher René Moineau idealized the principle of progressive cavities. However, not having the financial resources to develop his project, Moineau sold his patents to some companies. His principles resulted in the progressive cavity pumping (PCP) lifting system, which is nowadays the main technology for oil well production in the world^1^.

**Figure 1:**
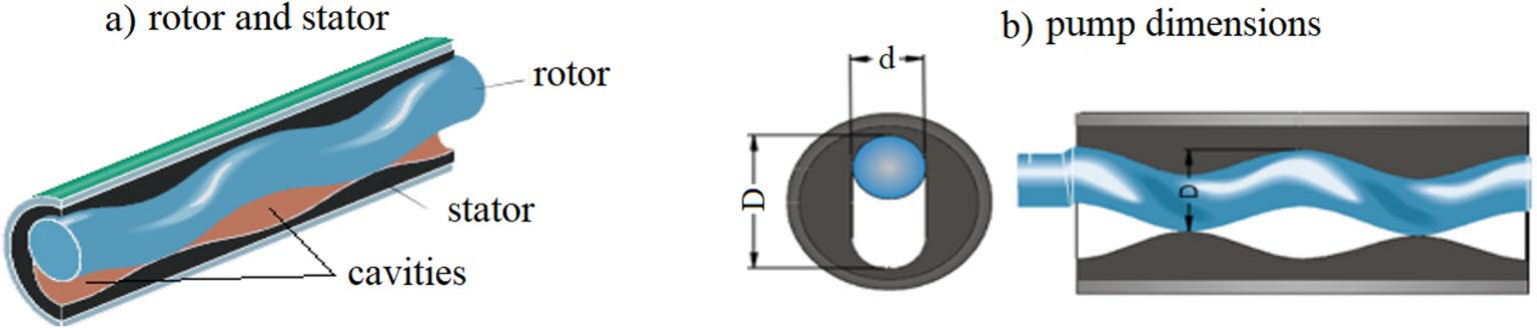
Rotor and stator with their dimensions: (**a**) Rotor and stator; (**b**) Pump dimensions. Source: Netzsch internal specifications manual (modified).

The PCP artificial lift method consists of a progressive cavity pump installed inside the well at the lower end of the production column through which fluid is pumped^1^. In Fig. 1 (left), it can be seen that the progressive cavity pump basically consists of two components, a stator, and an impeller, and is driven by the rotation of the impeller through the rod column. In addition, it can be noted in Fig. 1 (right) the dimensions that are called major diameter (D) and minor diameter (d), both in the rotor and in the stator.

There are several works focused on designing electrical machines with better efficiency and development of fault diagnostics^2^, with applications optimizing the machine design through the finite element method (FEM)^3^ and artificial intelligence^4^. Increasingly, FEM-based approaches are being successfully applied for the development of equipment that is more robust^5–7^. Even simple solutions can be used to improve the performance of electric motors by simply changing the way they are used^8^. On the other hand, artificial intelligence methods are being improved to obtain models with greater capacity^9^. In this context, fault diagnosis can be performed by means of time series analysis^10–12^, computer vision^13^, or general pattern recognition^14^.

During the rotation movement of the rotor, voids are formed and sealed in the cavity (Fig. 1) of the stator, which is filled by the fluid to be pumped. With the rotation of the rotor, these voids move continuously and progressively in the direction of the helicoid pitch, dragging the fluid in the direction of the pump discharge. It is noteworthy that the ability to generate pressure difference of the PCP is controlled by a combination of the maximum pressure of each cavity and the number of cavities. How much pressure difference each cavity can generate is related to the ability to create seal lines between the rotor and stator and the properties of the pumped fluid. This seal line is improved according to the interference between the rotor and stator, that is, the smaller the space created between them^15^.

The creation of the seal line between the rotor and the stator happens by combining their dimensions (D and d), with this it is possible to obtain greater success in the result of the performance test on the bench, ensuring a good useful life of the pump in the well. This combination is called pump sizing. The lifetime of a PCP is determined by the interaction between the rotor and stator in the sealing lines (interference) that are controlled by the pump sizing. The pump sizing process is very complex and requires rigorous studies on pump behavior on test benches, fluid composition, and characteristics of the well where the pump will operate, among others. To simplify this process, many PCP manufacturers usually manufacture rotors with different dimensional ranges in the smallest diameter (d) and largest diameter (D) of the rotor for each pump model. These ranges are categorized as standard, single, double oversized, or undersized^16^.

Based on the arguments exposed above, this paper presents the construction of a prototype based on graph neural networks (GNNs) that performs the interference recommendation and, as a result, calculates the rotor size so that it, combined with the stator can achieve the acceptance criteria obtained during the bench performance test. In addition, it is intended to simplify all the steps performed during pump sizing by increasing the number of examples that can be used as reference, resulting in a more assertive definition.

The use of GNNs is becoming increasingly popular due to their high capability in dealing with data that has inter-sample connections. From this property, it is possible to classify and/or predict the conditions of a sample and its class, based on its interaction with other samples. These interactions are the connections that generate the graphs, when samples are connected with other samples they are called nodes in the graphs. Several graph-based structures have been explored and many variations in their nomenclature and application are presented^17^.

The great advantage of using GNN is that models based on this concept have the ability to be trained with respect to the relationships between connections to other samples^18^. GNNs predict higher chances of a node belonging to a class in relation to the connections that this node has with nodes of the same class. Since there are variations in the structure of the GNNs next section presents a discussion about related works considering these variations.

## Related works

The development of deep learning models is growing due to improved processing capabilities in hardware nowadays. The use of data represented in the Euclidean space is common to evaluate using these models, however, new approaches are being used from data from non-Euclidean domains^19^. These data-represented graphs have high complexity because they have connections beyond the basic features that are evaluated in classification problems. The use of deep learning for GNNs is a promising alternative for solving tasks in several fields such as biomedical^20^, transportation^21^, energy^22^, and security^23^.

According to Wu et al.^17^, GNNs can be divided into 4 categories, being recurrent GNNs, convolutional GNNs, graph autoencoders, and spatial-temporal. To improve the semantics extraction capability of convolutional neural networks (CNNs), which are currently widely used for image processing in computer vision^24, 25^, Jiang et al.^26^ proposed a method that combines CNNs and GNNs for fusing multi-level features, and this combination is shown to outperform well-established methods.

In graph convolutional networks (GCNs) there are many ways to employ convolutional layer operators. Based on these variations alternative models are proposed such as the graph transformer network^27^, graph isomorphism network^28^, multi-attention label relation learning CNN^29^, causal incremental GCN^30^, principal neighborhood aggregation-based GCN^31^, among other approaches. Since GCNs do not assign different weights to neighboring nodes, graph attention network (GAT) gains ground as it takes different neighboring nodes into account^32^.

Several models require all nodes of the graph to be present in the training process of the embeddings, approaches that do not generalize invisible nodes. According to Hamilton, Ying, and Leskovec^33^, the GraphSAGE (SAmple and aggreGatE) model uses the node feature information to generate the node embeddings for previously unknown data. Thus, instead of learning a function, GraphSAGE generates embeddings by sampling and aggregating features from the local neighborhood of a node.

Models that combine techniques such as graph convolutional recurrent neural networks (GCRNN) have been explored. According to Ruiz, Gama, and Ribeiro^34^ when compared to GNN models GCRNNs perform better because they use fewer parameters. These models use convolutional filters to keep the number of trainable parameters independent of the size of the graph. In addition to the classification evaluated in this paper, also prediction^35^ and semantic segmentation^26^ using GCRNN are becoming popular.

Besides the convolutional layers GCN, GAT, and GraphSAGE, other types are proposed and can be used to create the GNN models. Table 1 shows examples of studies involving GNNs in different applications, for comparative purposes, prediction and forecasting are considered equivalent. All these approaches discussed about convolutional layers, in addition to the GCRNN, can be used for both classification and prediction as can be seen in Table 1.

**Table 1:** Related works.

Authors	Method	Application
Li et al.^36^, Bai et al.^37^	GCN	Traffic prediction
Zhao et al.^38^		
Li et al.^39^, Jiang et al.^40^	GCN	Disease prediction
Parisot et al.^41^		
Zhou et al.^42^, Zhu et al.^43^,	GCN	Sentiment classification
Zhao et al.^44^		
Jiao et al.^45^, Li et al.^46^	GAT	Link prediction
Grassia and Mangioni^47^		
Yu et al.^48^	GAT	Weather prediction
Aykas and Mehrkanoon^49^		
Yang et al.^50^, Linmei et al.^51^	GAT	Text classification
Liu and Gou^52^		
Chen et al.^53^	GraphSAGE	Energy prediction
Chen et al.^54^		
Liu et al.^55^, Belle et al.^56^	GraphSAGE	Fraud detection
Jing et al.^57^		
Yao et al.^58^, Ding et al.^59^	GraphSAGE	Image classification
Zhou et al.^60^		
Elbasani et al.^61^	GCRNN	Protein prediction
Elbasani and Kim^62^		
Zhang et al.^63^, Xu et al.^64^	GCRNN	Traffic prediction
Ya et al.^65^		
Ruiz et al.^34^	GCRNN	Environmental forecast
Le et al.^66^		

## Proposed method

This paper has been established in accordance with the steps of the CRoss Industry Standard Process for Data Mining (CRISP-DM) model^67^, starting with the phase of understanding the problem and its data, the selection and treatment of data until a solid and reliable database is achieved, the definition of possible techniques to be used in the construction of the interference recommendation, and finally the evaluation of the results.

In the stages of business understanding and data understanding, it is necessary to follow up with the engineers who design progressive cavity pumps that perform the pump sizing process. In this step, an understanding of the process and why it is important during the manufacturing of progressive cavity pumps is obtained. In addition, all the tasks that are evaluated during the process are documented. Table 2 shows the 8 main tasks that are performed with each new input.

**Table 2:** Description of tasks performed in the pump sizing process.

No.	Task
01	Collect data of inlet pump test conditions
02	Search for similar pumps in the database
03	Search for the rotor and stator dimensions of similar pumps
04	Analysis of the rotor and stator dimensions of similar pumps
05	Interference calculation
06	Search for the current stator dimensions of the stator of the same pump model
07	Analysis of the stator dimensions of the pump model
08	Calculation of the rotor dimensions using the interferences

During the process documentation stage, the variables that directly, and indirectly influence the test results of progressive cavity pumps are relevant to pump sizing, and also are cataloged. With this, a total of 38 variables are obtained, among them are variables such as the pump model, test results, test criteria performed, and the complete dimensionality of the rotor and stator assembly that are linked to the test results obtained.

Currently, most of the variables are available in expert systems (non-integrated) with their own database, and another part is in spreadsheets used by the design engineers. Given this scenario, it is decided as a starting point to select data from the system that stores all performance tests of progressive cavity pumps, called performance curves. In this selection the pump serial number, order confirmation number, rotor code, test date, stator surface temperature, test fluid temperature, and stator part code from the year 2019 are considered resulting in 8,714 test records.

The data obtained from this selection went through the cleaning process, as they presented typing errors and a lack of specific fields within the system. In addition, data related to the stators underwent a transformation in its original structure, because stators can be built from one or more welded parts. In this case, the information stored for each complete stator had to be replicated, where each line represents a stator part that makes up the complete stator.

Once the data had been organized and processed, it is used to make a new selection, adding data regarding the test conditions of each order confirmation that are stored in the enterprise resource planning (ERP)^68^. In this selection, information is added to the data, such as the fluid used in the test, the rotation and pressure by which the test results will be evaluated for the pump acceptance, and the limits of volumetric efficiency defined by the customer, which the pump must have to obtain acceptance. Finally, a dataset with 48,276 records and 37 columns is obtained, representing the entire path from the conception of the sales order for the pump to the final test on the test bench.

**Figure 2:**
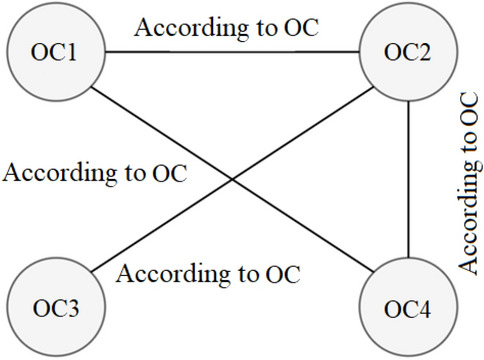
Network representation. Source: Drawn by the author.

To use an approach similar to the process done manually, in which historical data is used to perform pump sizing, in this paper it is chosen to use complex networks to evaluate the influence that an order confirmation (OC) has in relation to the others. In modeling the network, it is considered it non-directed, because both vertices connected by edges are considered similar, and both can be used as a reference to each other. Figure 2 shows a sample network in which each vertex represents an OC and each edge represents one or more reference(s) to other order confirmations. From the relation of each OC a graph is built.

### Graph neural networks

Graph-based problems have a large application because of the connections between nodes that can improve the classification ability of the model, as they are additional information that can be learned and help in the classification task. GNNs capture the dependency of graphs by passing messages between graph nodes. These techniques become more popular as information becomes more connected, especially among Internet users. In addition to Internet applications, GNNs can be used in many fields where there are connections between samples that may help in the classification task^69^.

Graph neural networks have generalization capabilities and because of their underlying relationships, they can be applied in various fields of science and engineering^70^. A graph ($G_i$) is a pair of nodes ($N_i$) and edges ($E_i$) that may have related features that result in information that can be used to classify or predict conditions. Learning in GNNs consists of estimating the weight parameter, such that it approximates the data from the learning data set (${{\mathcal {L}}}$):

1 $$\begin{aligned} \begin{matrix} {{\mathcal {L}}}=\{({G}_{i},n_{i,j}, {t}_{i,j})\vert , {G}_{i}=({N}_{i}, {E}_{i})\in {{\mathcal {G}}}; n_{i,j}\in {N}_{i}; \\ {t}_{i,j}\in I\!\!R^{m}, 1\le i \le p, 1\le j \le q_{i}\} \end{matrix} \end{aligned}$$

where $n_{i,j}$ denotes the $j_{th}$ node, $t_{i,j}$ is the desired target associated to $n_{i,j}$, and $q_i$ is the number of supervised nodes in the graph^71^.

The use of graph-based approaches is growing in many applications, there are several models that have been successfully presented for the classification task. Among these models, the GCN proposed by Kipf and Welling^72^ and the GAT presented by Veličković et al.^73^ are standing out due to their performance compared to other classifiers.

**Figure 3:**
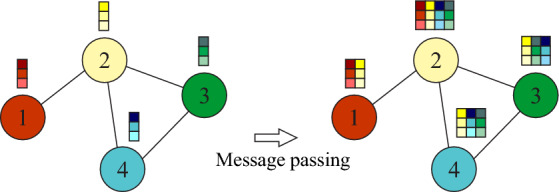
Nodes receiving the message per adjacent node. Source: Drawn by the author.

In GCN there are several ways to use operators in convolutional layers. The GCN model is based on convolutional networks which through a scalable approach is efficient for solving graph problems. In GCNs each node creates a feature vector, which represents the message that should be sent to its neighbors^72^. Messages are sent in a way that a node receives one message per adjacent node, this passing of the message between connections is represented in Fig. 3.

According to Kipf and Welling^72^, the GCN is promising because it is based on the number of graph edges and the features of each node. This method is validated in several benchmarks for classifying nodes in very popular citation datasets, like Cora, Citeseer, and Pubmed. The GNC can be extended to other applications such as remote sensing^74^, fault diagnosis^75^, emotional action recognition^76^, traffic prediction^38^, and image classification^77^.

Several frameworks have been used to solve graph-based problems, such as graph attention networks^78^, graph convolutional networks^79^, heterogeneous graph neural network^80^, heterogeneous graph attention network^81^, and so on. As well as classic neural network models^82^ or models based on deep learning^83–85^, the choice of the appropriate framework is critical for successful application.

**Figure 4:**
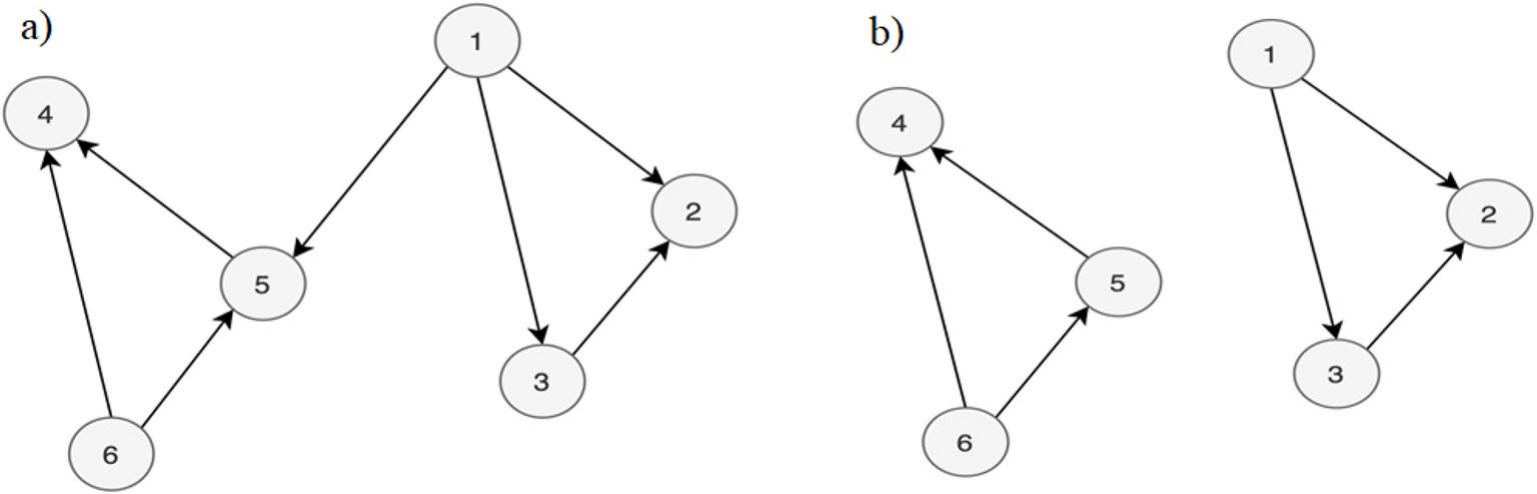
Graph: (**a**) Connected components; (**b**) Disconnected components. Source: Drawn by the author.

With the network modeled, the algorithms are applied for identifying connected components and calculating the betweenness centrality metric. A graph *G* (Fig. 4a) is connected if there is a path connecting each pair of vertices of *G*, otherwise *G* is said to be disconnected. Thus, a disconnected graph has at least two connected subgraphs, called connected components (Fig. 4b).

The GNNs are being most widely used for their ability to extract additional information from the data^70^, in addition to GNNs, deep learning-based models are constantly being explored for their ability to learn nonlinear patterns^86^. Models that use ensemble aggregating weaker learners also have their place within this context^87^.

**Figure 5:**
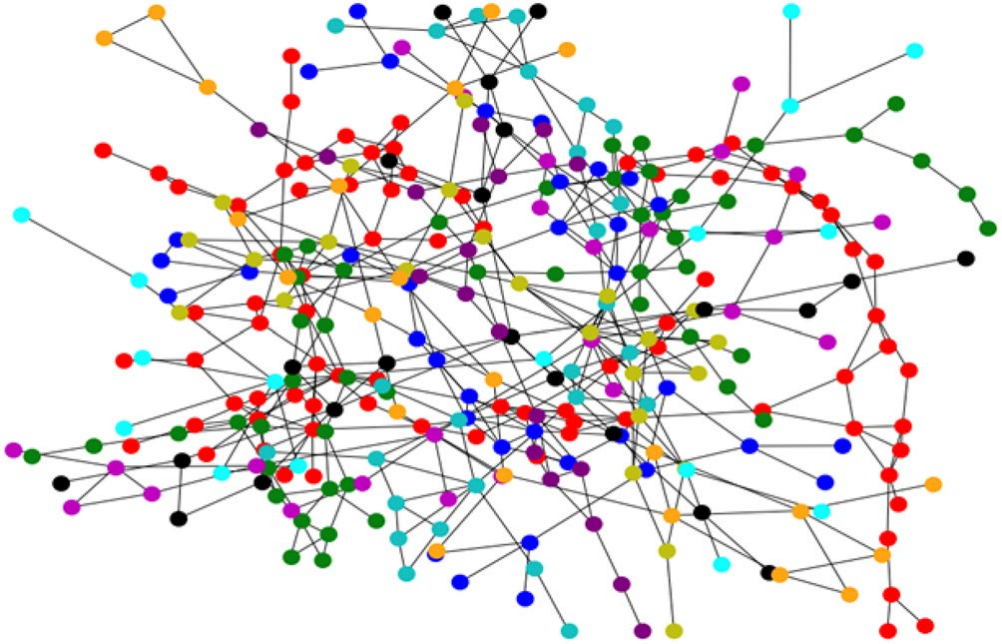
Connected components. Source: Author’s simulation results using Python.

The search for connected components returns a SET Generator with the OC of the connected component. Identifying the connected components in this context is important for clustering OCs that are used as a reference to perform pump sizing. Also, consequently, these connected components have a similarity in their technical characteristics, test criteria, and test results in their OCs and can bring additional information to the analysis. In Fig. 5 it is presented a plot of ten OCs of the graph (each OC has a different color).

With the related components defined, the betweenness centrality metric are calculated, determined by Eq. (2).

2 $$\begin{aligned} cv(v)= \ \sum _{s,t\ \in \ V}\frac{\sigma (s,t \mid v)}{\sigma (s,t)} \end{aligned}$$

where *V* is the set of vertices of the graph, $\sigma (s,t)$ is the number of paths between vertices (*s*, *t*), and $\sigma (s,t\mid v)$ is the number of these paths that pass through some vertex *v* other than (*s*, *t*), if $s=t,\sigma (s,t)=1$ and if $v \in s,t,\sigma (s,t\mid v)=0$.

This metric is used to measure which OC has the most influence on the connected component it is part of. The highest values of this metric are used as a recommendation and its data is used for pump sizing. The return is a dictionary where the key identifies the OC number.

**Figure 6:**
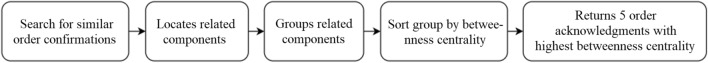
Steps for interference recommendation. Source: Drawn by the author.

At the end of this process, there is a list of dictionaries where each dictionary is a connected component containing the OC number as a key and the betweenness centrality value. With the related components defined and each OC having its betweenness centrality calculated, the step that performs the interference recommendation for the incoming OC begins. Figure 6 shows the main actions performed to make the interference recommendation.

Initially, a search for OCs is applied to the data set considering the pump model, stator rubber, and pump test parameters, and then the related components to which each OCs is part are located, such connected components are grouped and ordered by the value of the betweenness centrality metric. With this, there is an ordered group of connected components that have similarity with the input. From this, the first 5 OCs with the highest value of betweenness centrality, that is, with the greatest influence, are selected.

Based on the five recommended OCs, their clashes are used to define the new clash. It is chosen to use the median central tendency measure for this definition, because it is known that there may still be outliers between the recommended cases and they can negatively influence the value of the new interference when the average is used.

To calculate the rotor dimensions, measurements *D* and *d* of the stator are necessary. In the current process, the stator dimensions are monitored to ensure that the dimensions are close to the stator dimensions of the recommended cases. This monitoring is necessary because changes in the process, and changes in the rubber formulation, among others, can cause changes in the stator dimensional. It is noteworthy that the dimensions of the last thirty stators manufactured are used, calculating the average of the *d* of these cases.

To search for these stators, parts of the data that make up the pump model and the type of stator rubber are used. The pump pressure is disregarded because it is not a variable that influences the stator dimensional change. Finally, the rotor dimensions are calculated, applying the stator dimension found and the recommended interference.

It is worth mentioning that, to calculate the rotor measurements, the most relevant dimensions and the ones that most influence the result of the bench test for each type of geometry are considered. The rotor diameter measurement (*dr*) of Single-lobe pumps are calculated using only the smallest diameter of the stator (*d*), according to:

3 $$\begin{aligned} dr=d+i. \end{aligned}$$

where *i* is the interference. For Multi-lobe geometries the largest diameter (*D*) is also considered, and then the *dr* is:

4 $$\begin{aligned} dr=msd+i. \end{aligned}$$

where *msd* is the mean stator diameter, given by:

5 $$\begin{aligned} msd=\ \frac{(D+d)}{2}. \end{aligned}$$

## Results analysis

To test the proposed model, the data set with 48,276 records and 37 columns, resulting from the data selection and cleaning steps, is used. From these data, only the tests where the acceptance rotation is equal to the test rotation are considered, because the pump is tested in several rotations, in this case, only the data of the rotation of interest to which the pump will be evaluated is desired. Therefore, there is a reduction in the data set to 11,673 records.

These data, in turn, are divided into two parts, a part called training, with 80% of the records, which is used to build the network, and the second part called test, with 30% of the records, which are used as input for the recommendation. With the network constructed from the training data, the tests for the recommendation can be performed using the test data as input. In this step variables described in Table 1 categorized as input are considered.

To evaluate the recommendations, it is decided to compare the average dimension *d* of the impeller in pumps with single-lobe geometry or the average dimension *d* of the impeller in pumps with multi-lobe geometry with their respective real average dimension. In Table 3 there is a sample of the input variables as well as the target variable y_true which is the real rotor dimensional and which will be compared with the recommended rotor dimensional y_rec defined by the recommendation.

**Table 3:** Input data and target variable.

Variable	Value	Type
type_tube	NTZ	Input
nominal_pipe_diameter	400.0	Input
pump_geometry	ST	Input
pump_flow	62.0	Input
high_pressure	YES	Input
rubber_stator	286.0	Input
test_fluid	0.0	input
temp_fluid_test	50.0	Input
temp_up_stator_test	30.0	Input
rpm_acceptance	300.0	Input
rpm_test	300.0	Input
pressure_acceptance	180.0	Input
efficiency_min	0.0	Input
eff_max	0.0	Input
y_true	41.48	Target
y_rec	41.26	Recommendation

A total of 2,972 tests are performed involving different pump models and test criteria. The first evaluation made on the recommendation is to quantify the number of cases in which it is successful in making recommendations. Within the 2,972 tests, 69% of the cases had recommendations, this happens because the recommendation did not find any OC similar to the given inputs.

Next, the quality of the recommendation is checked, for this, the mean squared error (MSE) metric is used, which calculates the difference between the real rotor measure and the recommended rotor measure, high MSE values, indicate that the recommendation did not perform well in relation to the recommendations. Applying this metric to the recommended data obtained a score of 0.28. It is worth noting that, to calculate the MSE metric, the cases in which there are no recommendations are removed from the recommendation dataset, i.e., the MSE is calculated using 69% of the cases that had recommendations.

In addition to the MSE metric, a check is made on recommendations that had a dimensional difference between y_true and y_rec greater than 0.5mm. With an error less than or equal to 0.5mm it is still possible to do rework on the rotor helicoid, this rework consists of adding a layer of hard chrome over the helicoid or removing it by sanding. Recommendations with differentials greater than 0.5mm affect the profile of the rotor helicoid, which in turn affects the sealing between the cavities. In this context, 6% of the cases tested showed a dimensional difference between y_true and y_rec greater than 0.5mm and 94% less than 0.5mm.

**Figure 7:**
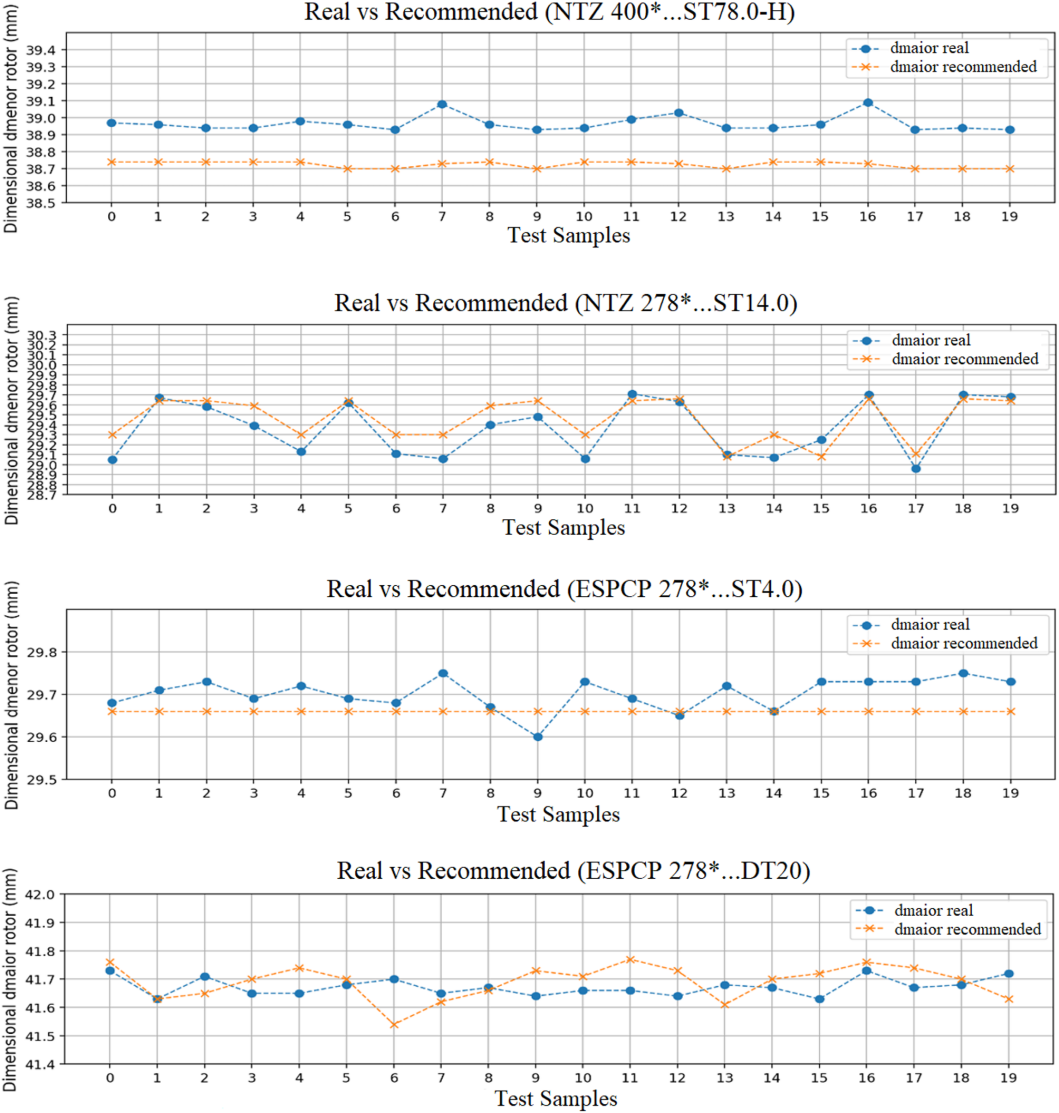
Real vs. recommended dimensional comparison. Source: Author’s simulation results using Python.

Lastly, visual sample checks are performed by grouping the test results by pump model, except for pump pressure. Figure 7 shows in graph form 20 samples of different pump models comparing their real dimensions with the recommended dimensions.

It is possible to see a similarity between the real dimension and the dimension recommended by the recommendation. The dimension of the rotor represented by the *y* axis shows a decimal variation between the dimensions, in some cases, the precision is even lower, and in others the recommendation is identical to the real dimension.

Although there is a variation between the real dimension and the recommended dimension, it is worth pointing out that this variation is very small and considered acceptable for this context, since to calculate the dimensions of the rotors the stator measures are used, which can suffer dimensional variations that are reflected in the calculated dimensions of the rotor. Besides this, in the current process, there is a cycle time of approximately 62 minutes to perform the pump sizing of each OC. With the implementation of the proposed method, it is estimated the reduction of this time, for cases in which there are recommendations, is about 50% for each OC for which the pump sizing is done.

### Comparison to results of other authors

In the work of Zhu et al.^88^ an MSE of 0.016 was achieved in a communication-related problem, in which a GNN-based model was proposed for improving network performance by utilizing multiple paths simultaneously. By leveraging the power of GNNs, their model optimizes network performance, ultimately enhancing the user experience and the overall efficiency of multipath communication protocols.

In the research of Numcharoenpinij et al.^89^ using embedding-GNN they had an MSE of 174.952 for predicting drug interaction. This shows that there are cases where the challenge is more complex, and thus the results of MSE may be higher.

Comparatively, in^90^ an MSE value of 3.38 was obtained for the estimation of traffic speed. Using the GNN, Zuo et al.^91^ had an MSE of 0.9804, and Wang, Hu, and Zhang^92^ had an MSE of 0.217, both considering an evaluation of drug response prediction.

In this paper the MSE of 0.28 was achieved, this result has a great influence on the characteristics of the data, which in some cases may have fewer related and non-linear patterns, which means that the results may be inferior or in some cases superior when the task is simpler to solve.

## Conclusions

This work presented a method for building an interference recommender, helping users in decision-making and accelerating steps performed during the pump sizing process. Besides the interference recommended, it is possible to diagnose the data used by the recommender, so that an evaluation can be done by experts to try to explain the recommendation, even in cases where the recommendation is not aligned with the user’s definition. Thus, with the help of the recommender, besides delivering a recommendation, the amount of data analyzed by it is much larger if compared to the amount of data that is manually analyzed by the user in the current process.

During the data selection and cleaning stage, several problems related to data quality are identified, due to some legacy systems, which have not been updated during the change of processes over the years, and the use of Excel tables where, both scenarios, give users the freedom to register information without any kind of standardization. In this context, it is decided to eliminate the doubtful records or those with empty fields, impoverishing the database used in this work.

The use of complex networks proved to be very assertive; the construction of a graph represented very well the current analysis model where, during the pump sizing process, previous OCs are used as a reference. In this scenario, OCs are represented by vertices and edges indicating references to other OCs. This allows you to identify the connected components that helped you find similar OCs among the data. In addition to the connected components, the betweenness centrality metric can be used to measure the importance of each vertex in the graph, using this measure to recommend the most important OCs to the user and that consequently are the most used in past definitions.

With this, in the tests performed, the method proved to be very efficient. The recommendation index close to 70% already represents a considerable reduction of user time when performing pump sizing, even if the result does not agree with the user’s opinion, the user can use the data selected by the recommendation to evaluate the results. In addition, the MSE metric reached a value of 0.28 validating the closeness of the recommended dimensional to the real dimensional measured on the rotor.

A sample comparison of the recommended and real dimensional values showed acceptable recommendations to start testing in a production environment with experts following up to perform a final evaluation. Despite achieving good results in a test environment, it is worth mentioning that it is possible to achieve better recommendation levels by enriching the database, including new variables for verification, implementing new pump model similarity algorithms, and performing a seasonal decomposition on the data, among others.

## Data Availability

The data will be made available upon request to Leandro Starke.
